# A Commentary on “Medical learning: From clinical-to-tech-based thinking (beyond typical clinical signs)”

**DOI:** 10.1016/j.amsu.2021.102717

**Published:** 2021-08-12

**Authors:** María Gabriela Ascencio-Vera, Carlos Iván Higuera-Cetina, Melissa Charria-Caicedo, Víctor Andrés Aguirre-Orjuela, María Paz Bolaño-Romero, Sabrina Rahman

**Affiliations:** School of Medicine, Universidad de La Sabana, Carrera 69 # 80 - 45, Chía, Colombia; School of Medicine, Universidad de Boyacá, Carrera 2^a^ Este No. 64 - 169, Tunja, Colombia; School of Medicine, Fundación Universitaria San Martin, Cra. 21 #25-39, Cali, Colombia; School of Medicine, Fundación Universitaria Juan N Corpas, Cra. 111 #157-61, Bogotá, Colombia; Medical and Surgical Research Center, School of Medicine, University of Cartagena, Cra. 50 #24-120, Cartagena, Colombia; Department of Public Health, Independent University- Bangladesh, Dhaka, Bangladesh

Dear Editor,

We have read with great interest the article published by Mohammad AM [1] titled “Medical learning: From clinical-to-tech-based thinking (beyond typical clinical signs)”, where the author comments on an interesting aspect of medical education, proposing the transition from the traditional clinical approach, to the approach of robotic technologies and tools for early diagnosis and improvement of disease burden, implementing this thinking from the undergraduate level [1]. We thank Mohammad AM [1] for his perspective. However, we consider relevant to expose some comments about the determinants that currently delay the progress of such an educational model, and the possible strategies to implement to achieve a significant and favorable change in undergraduate medical education.

Today, in post-pandemic times, there are many determinants that define quality medical education, specialty choice and professional success in medicine [[2], [3], [4], [5], [6], [7]]. Participation in medical interest groups to develop specialized skills and deepen theoretical and practical concepts according to the area of interest, work policies, salaries and lifestyle of the surgical and medical specialties of each country, as well as the workload of care, define how favorable and resilient will be the change in the organization and decision making in medicine [[2], [3], [4], [5], [6], [7]]. However, in order to transcend the traditional dual educational models, which focus only on lectures in classrooms and practices in medical rounds, it is also necessary to know the evidence and be intimately related to new technologies and the objectives of global health in the short-, medium- and long-term.

Studies such as Motte-Signoret et al. [8] and Gismalla et al. [9], which have investigated the perception of medical students and teachers on the change of medical education towards virtuality, have found heterogeneous perceptions on the use and impact of this strategy [8,9]. Motte-Signoret et al. [8] showed that less than half of the learners and teachers perceive that they are adequately trained in the use of virtual educational tools, which affects their attitude towards the classes given and received [8]. In low- and middle-income countries, Gismalla et al. [9] found that out of 358 students surveyed, two-thirds agreed with the implementation of virtualization in medicine, despite encountering barriers such as limited connectivity and quality of broadband internet, unfamiliarity with educational tools, limitations on technical support and availability of quality computers, which hinder the development of quality learning [9].

But beyond that, there is one factor that is evident in the daily practice of medical education that has a substantial impact on the relevance of medical education, and that is the continued presence of older professors who practice and believe rigidly in experience-based medicine, rather than evidence-based medicine. Those teachers who consider medical semiology as a whole, and want to put aside the technological tools that facilitate the medical practice. This problem is seen with greater intensity in third world countries, where there is not the same research development on medical education and design of evaluation instruments [10], reducing the interaction with recent evidence in medical education. For example, Hosny et al. [10] developed, validated and implemented a tool to measure teacher preparedness for e-learning during the COVID-19 pandemic, noting that of five variables assessed (Online Teaching and Course Design Skills, Digital Communication, Basic Computer Skills, Advanced Computer Skills and Using Learning Management Systems), Online Teaching and Course Design, and Using Learning Management Systems are the ones most in need of improvement [10]. However, it is worth asking, is it possible that a professor who is not familiar with the virtual tools of medical education will possess or develop skills for the variables mentioned above, which require specific postgraduate preparation?

To improve the functional outcomes of medical schools globally, but especially in low- and middle-income countries [11], it is necessary, first, to require that professors be specialists in education (and possibly in medical education), and second, that they have an intimate relationship with evidence and technological tools in medicine, since it can be presumed that only they will have the ability to propose solutions to any crisis such as the one presented during the COVID-19 pandemic. The permanence of a large number of very old teachers could be a factor to consider in this situation.

## Data statement

Data sharing is not applicable to this article as no new data were created or analysed in this study.

## Ethical approval

Not applicable.

## Sources of funding

None.

## Author contribution

All authors equally contributed to the analysis and writing of the manuscript.

## Research registration Unique Identifying number (UIN)

1. Name of the registry: Not applicable.
2. Unique Identifying number or registration ID: Not applicable.
3. Hyperlink to your specific registration (must be publicly accessible and will be checked): Not applicable.

## Guarantor

S.R. Department of Public Health, Independent University- Bangladesh, Dhaka, Bangladesh -

## Provenance and peer review

Commentary, internally reviewed.

## Declaration of competing interest

All authors declare that there exist no commercial or financial relationships that could, in any way, lead to a potential conflict of interest.
